# Sternal Reconstruction for Refractory Pectus Excavatum From Facioscapulohumeral Muscular Dystrophy

**DOI:** 10.1016/j.atssr.2024.06.006

**Published:** 2024-06-25

**Authors:** Daniel Kyrillos Ragheb, Sigrid Johannesen, Erin Gillaspie

**Affiliations:** 1 Department of Thoracic and Cardiovascular Surgery, Heart, Vascular and Thoracic Institute, Cleveland Clinic, Cleveland, Ohio; 2 Department of Thoracic Surgery, Vanderbilt University Medical Center, Nashville, Tennessee

## Abstract

A 44-year-old man with a history of facioscapulohumeral muscular dystrophy and pectus excavatum presented with multiyear history of progressive, severe respiratory dysfunction, pain, recurrent respiratory infection, and chest wall deformity. With bioprosthetic engineers, the surgical team customized a 3-dimensional printed model of a sternal implant interacting with the patient’s anatomy. After approval from the Food and Drug Administration, the customized sternal plates were created and implanted in a sternal reconstruction operation. We report on the successful implantation of a customized sternal plate in the treatment of a patient with refractory pectus excavatum in the context of facioscapulohumeral muscular dystrophy.

Facioscapulohumeral muscular dystrophy (FSHD) can be manifested with respiratory insufficiency, particularly in the setting of both bone deformity and severe muscle weakness. A portion of these patients report severe deterioration of respiratory function.^1^ Sternal reconstruction may be a therapeutic option, but it is most commonly performed in the setting of sternal resection for cancer, trauma, infection, or severe deformity; it is less well described in the FSHD population.

The use of customizable hardware is relatively new in sternal reconstruction. Previous authors, such as Kamel and associates,^2^ reported the use of 3-dimensional modeling and customizable implants in the repair of sternal composition in patients who presented with reduced respiratory function. However, these authors reported on the use of this operative technique to treat respiratory dysfunction secondary to sternal chondrosarcoma and paradoxical anterior chest wall movement, not severe or recurrent pectus excavatum. We present a case of severe pectus excavatum in the setting of FSHD and the use of 3-dimensional modeling and customizable implants as a therapeutic option to reduce respiratory distress, pain, and chest wall deformity.

A 44-year-old man with severe pectus excavatum (Haller index, 4.9) and underlying FSHD presented with a multiyear history of progressive, severe respiratory dysfunction, pain, recurrent respiratory infection, and chest wall deformity. He was previously denied surgical intervention because of his age and underlying disease. He ultimately underwent a Ravitch operation with concomitant scapulothoracic fusion to enhance support of the chest wall but experienced subsequent loss of sternal projection due to fibrous replacement of the pectoralis muscles and progressive weakness of the shoulders and upper arms.

The first operation, a Ravitch, was performed 2.5 years before presentation. The xiphoid process was resected, an osteotomy was created below the manubrium, and the sternum was moved 3.5 cm anteriorly. The sternum was then plated in place. Postoperatively, the patient was satisfied with the physical appearance of his surgery and had marked improvement of respiratory function. The patient underwent a second orthopedic procedure, scapulothoracic fusion, in which the anterior scapula and posterior thorax are fused to provide further stabilization. After completion of both operations and despite short-term improvements and continuous bracing, the patient subsequently lost about 3 cm of sternal projection with only partial fusion of the shoulder and an inability to proceed with the contralateral side.

The patient presented to our center with progressive shortness of breath and activity limitation. On evaluation of pectus excavatum, preoperative pulmonary function test results showed a restrictive pattern with a forced expiratory volume in 1 second/forced vital capacity ratio of 89% and a forced vital capacity at 59% of predicted value. Preoperative echocardiography demonstrated no cardiac concerns, with no compression of the heart and normal left ventricular function. Our surgical team, in collaboration with bioprosthetic engineers, created customized plates that would leverage the patient’s midclavicular rib cage, which had sufficient projection and stability to support the sternum without reliance on musculature. A customized 3-dimensional printed model of the implant and the patient’s anatomy was created to trial and rehearse the approach as well as to provide comprehensive patient education. Approval from the Food and Drug Administration was obtained for the custom implant.

A submammary incision was created for access, using the previously created incision with bilateral extension. The sternum was freed from adjacent calcified perichondrium and scar tissue. A sternal plate was identified and resected. The sternum had healed into an inadequate position, and thus a new wedge osteotomy was performed, allowing the sternum to be rotated anteriorly and 6 to 7 cm anteriorly. The second through fifth ribs were carefully exposed bilaterally. With slight modifications, the customized sternal implant fit perfectly along the patient’s ribs and secured the sternum in its new position (Figure 1).

**Figure 1:**
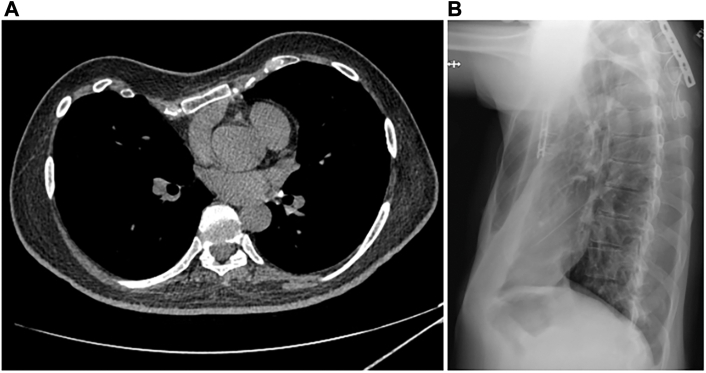
Preoperative imaging. (A) Computed tomography scan showing loss of sternal projection. (B) Chest radiograph showing pectus excavatum.

A bioprosthetic mesh sling was then secured to the sternum, ribs, and rectus abdominis musculature inferiorly to reconstruct the inferior chest wall. Postoperatively, the patient was immediately admitted to the step-down unit. He reported excellent pain control, depth of respiratory effort, and satisfaction with the improved chest wall configuration. He was discharged on postoperative day 6, and discharge chest radiography (Figure 2) demonstrated intact hardware with excellent pulmonary expansion. At 4-month follow-up, the patient continues to report excellent respiratory function, has returned to exercise, and reports minimal chest wall pain.

**Figure 2:**
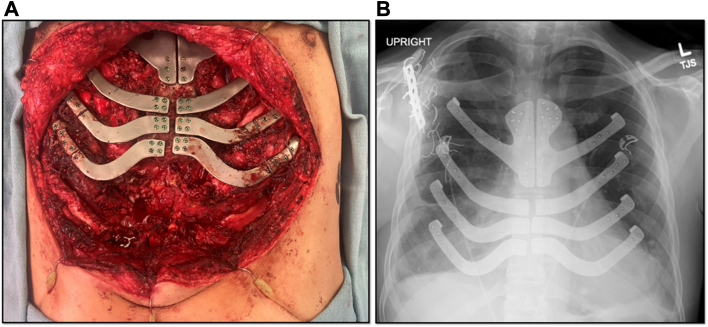
Final sternal plating. (A) Intraoperative view. (B) Chest radiograph on discharge.

## Comment

The use of customizable hardware is not entirely new to the thoracic field, although it remains rare in sternal reconstruction. We report on the use of customizable sternal reconstruction to treat refractory pectus excavatum secondary to FSHD.

FSHD patients experience a slow loss of strength that can lead to severe deterioration of respiratory function over time.^1^ This can be further exacerbated by the presence of pectus excavatum. There are 2 leading theories as to why FSHD may result in severe pectus excavatum; one suggests that it is part of a genetic or metabolic syndrome, whereas another posits that weak pectoral muscles are not able to counteract negative pressure in the anterior chest space, resulting in repetitive mechanical stress ultimately leading to severe pectus excavatum.^3^^,^^4^ Our patient had severely limited musculature, especially of the pectoralis, favoring the second theory.

Repair of pectus excavatum in this population of unique patients may be undertaken; however, traditional approaches may not prove to adequately provide long-term benefits secondary to the lack of supportive chest wall and scapular musculature. A patient-specific, customized approach should be favored to achieve better chest wall configuration, and in this circumstance, we elected to design a custom chest wall plate to leverage the most stable portion of the chest, which was the upper ribs. The implant was designed in pieces to allow better customization and alteration as needed at the time of implantation and to allow sternal division should future chest surgery be required.

The patient has had excellent improvement in respiratory function, allowing an increase in physical therapy to help preserve his strength and delay the need for supportive orthopedic devices and wheelchair.

This case highlights the importance of a personalized approach to medicine and the use of custom chest wall implants to palliate progressive respiratory failure secondary to underlying musculoskeletal disorders. Contemporary technologic advancements with imaging, 3-dimensional printing, and multidisciplinary teams made of engineers and physicians uniquely provided care to a patient previously deemed inoperable.
